# Association between the Endothelial Activation and Stress Index and all-cause mortality in patients with chronic obstructive pulmonary disease

**DOI:** 10.3389/fmed.2026.1732176

**Published:** 2026-05-13

**Authors:** Yili He, Juntao Hu, Yang Li, Ling Huang, Wenyan Jiang, Zhiyi He

**Affiliations:** 1 Department of Critical Care Medicine, Guangxi Medical University Cancer Hospital, Nanning, China; 2 Guangxi Clinical Research Center for Critical Care Medicine, Nanning, China; 3 Department of Respiratory and Critical Care Medicine, The First Affiliated Hospital of Guangxi Medical University, Nanning, China

**Keywords:** 28-day mortality, 90-day mortality, COPD, critical care prognosis, endothelial activation and stress index, predictive performance

## Abstract

**Background:**

Endothelial Activation and Stress Index (EASIX) predicts outcome in diverse disorders; however, its independent association with short- to medium-term mortality in critically ill patients with chronic obstructive pulmonary disease (COPD) has not been examined.

**Methods:**

Data were drawn from the Medical Information Mart for Intensive Care-IV (MIMIC-IV) database and the Critical care datebase of Zigong Municipal Hospital, Sichuan, China. We identified consecutive COPD patients admitted between 2008 and 2019 in MIMIC-IV and between 2019 and 2020 in the Zigong cohort. EASIX was calculated from platelet count, creatinine, and lactate dehydrogenase measured within 24 h of ICU entry and log_2_-transformed. Multivariable Cox regression and restricted cubic splines assessed its relationship with 28-day and 90-day mortality after ICU admission; proportional hazards assumptions were verified. Subgroup analyses and interaction tests evaluated robustness. Discrimination was quantified by area under the receiver-operating-characteristic curve (AUROC).

**Results:**

We included 5,012 critically ill COPD patients from MIMIC-IV and 431 from the Zigong registry. In fully-adjusted models treating log_2_(EASIX) as a continuous variable, higher values were associated with increased 28- and 90-day mortality: MIMIC-IV: HR = 1.23 (95% CI: 1.17–1.28, *p* < 0.001) and HR = 1.18 (95% CI: 1.14–1.22, *p* < 0.001); Zigong: HR = 1.32 (95% CI: 1.17–1.50, *p* < 0.001) and HR = 1.32 (95% CI: 1.17–1.48, *p* < 0.001). After dividing log_2_(EASIX) into tertiles, we found that patients in the highest tertile faced significantly greater mortality than those in the lowest: in MIMIC-IV the 28-day hazard ratio was 1.66 (95% CI 1.39–1.98, *p* < 0.001) and the 90-day HR was 1.55 (1.33–1.80, *p* < 0.001), while in the Zigong cohort the corresponding estimates were 2.38 (1.41–4.00, *p* = 0.001) and 2.37 (1.47–3.81, *p* < 0.001). Log_2_(EASIX) showed a non-linear association with both 28- and 90-day mortality. In MIMIC-IV the inflection point lay at approximately 3.49; below this breakpoint the hazard ratio rose modestly (28-day HR = 1.168, 95% CI 1.100–1.239, *p* < 0.001; 90-day HR = 1.144, 1.088–1.202, *p* < 0.001), whereas above the breakpoint the risk increased sharply (28-day HR = 1.805, 1.367–2.383, *p* < 0.001; 90-day HR = 1.597, 1.219–2.092, *p* < 0.001). In the Zigong cohort the corresponding breakpoint was located between 3.42 and 3.70. Subgroups and interaction testing showed the log_2_(EASIX)–mortality link was stable. ROC analysis showed that log_2_(EASIX) predicts 28- and 90-day death with good accuracy, yielding AUROCs of 0.80 and 0.79 in MIMIC-IV and 0.83 and 0.83 in the Zigong cohort, respectively.

**Conclusion:**

Elevated log_2_(EASIX) independently predicts short- and medium-term mortality in critically ill patients with COPD.

## Introduction

Chronic obstructive pulmonary disease (COPD) was reported in 2010 to affect approximately 384 million adults worldwide, corresponding to a global adult prevalence of 11.7% that year (1). This heterogeneous lung disorder is characterized by persistent airway inflammation, emphysematous destruction of alveolar structures, and progressive airflow limitation (2); clinically, patients present with chronic cough, sputum production, and dyspnea. Diagnosis is established according to the Global Initiative for Chronic Obstructive Lung Disease (GOLD) criteria: a post-bronchodilator ratio of forced expiratory volume in one second to forced vital capacity (FEV₁/FVC) <0.70 on spirometry (2). While chronic airway inflammation is recognized as a central pathological feature (3), well-established risk factors and contributory mechanisms also include impaired lung development during childhood, concomitant or prior asthma, and post-infectious sequelae resulting from previous severe respiratory infections (4).

Inflammation associated with COPD can trigger systemic complications, most prominently in the cardiovascular system (5). A chronic state of persistent inflammation can lead to damage and dysfunction of the vascular endothelium (6). Endothelial cells lining the vessel wall are pivotal for maintaining multi-organ health and systemic homeostasis. In their healthy state, these cells dynamically regulate vascular tone, promote physiologic angiogenesis, mediate hemostasis at sites of injury, and provide an antioxidant, anti-inflammatory, and antithrombotic interface (7). Endothelial dysfunction, by contrast, manifests as blunted endothelium-dependent vasodilation, heightened oxidative stress, sustained chronic inflammation, increased leukocyte adhesion, and elevated vascular permeability (8). These findings provide indirect evidence supporting the association between endothelial injury and COPD. In contrast, the measurement of brachial artery flow-mediated dilation (FMD) to assess vascular responses to blood flow changes in pulmonary diseases has directly confirmed this link (9, 10). Oxidative stress and chronic inflammation—both of which are more commonly systemic in nature—can disrupt overall vascular homeostasis. In the context of COPD, these processes may impair vasodilation and compromise circulating stem cells, thereby hindering pulmonary vascular repair (11). This impairs pulmonary vascular remodeling and consequently exacerbates endothelial injury.

The Endothelial Activation and Stress Index (EASIX) was first investigated in hematologic disorders. In conditions such as transplant-associated thrombotic microangiopathy (TA-TMA) and complement-mediated atypical hemolytic uremic syndrome (aHUS), studies observed a coordinated pattern of rising lactate dehydrogenase and serum creatinine alongside falling platelet counts. This insight led to the integration of lactate dehydrogenase, creatinine, and platelets into a single continuous biomarker—EASIX (12). EASIX is considered an important marker of endothelial injury in hematologic diseases because, in independent cohorts, its level correlates positively with circulating NT-proBNP, soluble thrombomodulin (sCD141) (13), angiopoietin-2 (14, 15), and interleukin-18, and inversely with IGF-1 (16). The metric is now widely applied in hematologic research, including surveillance for thrombotic microangiopathy after allogeneic hematopoietic stem cell transplantation (16), survival prediction in myelodysplastic syndromes (15), detection of endothelial injury in CAR-T-cell recipients (17), and prognostication in diffuse large B-cell lymphoma (18). Subsequently, its use has been extended to predict endothelial injury in sepsis (19) and assess outcomes in atherosclerotic coronary artery disease, although the underlying mechanisms remain unclear (20). This suggests that EASIX may have broader clinical utility, potentially reflecting either a systemic stress state or underlying endothelial damage.

Currently, no studies have reported an association between the EASIX and outcomes in COPD. EASIX may, however, align with certain pathogenic mechanisms underlying COPD. We therefore hypothesize that EASIX can be used in the ICU to assess short- to medium-term prognosis in COPD and may serve as an early warning tool for risk stratification in critically ill COPD patients.

## Materials and methods

### Data source

This retrospective cohort study used the Medical Information Mart for Intensive Care-IV (MIMIC-IV, v3.1) database and the Zigong Municipal Intensive Care Database in China (21). The former maintained by the MIT Laboratory for Computational Physiology. The database contains de-identified data from approximately 300,000 patients admitted from 2008 to 2019. The author (Hé Yīlǐ, certification No. 56396864) completed the required CITI program and human research protection course to obtain access. The latter was developed and maintained by Zigong Municipal People’s Hospital, Sichuan Province, China, and captures 2,790 patients with severe infections admitted to the ICU from 2019 to 2020 in the intensive care database (22). All protected health information were anonymized per the HIPAA Safe Harbor Rule. The protocol was approved by the Ethics Committee of Guangxi Medical University Cancer Hospital (Approval No. KY2025800). We followed the STROBE guidelines for manuscript preparation.

### Patient selection

*Inclusion criteria*:

(1) ICU admission. (2) Age ≥18 years. (3) A diagnosis of COPD identified by the following ICD codes: 496, 49120, 49121, 49122, J440, J441, J449.

*Exclusion criteria*:

(1) For patients with multiple ICU stays, only the first ICU admission was retained. (2) ICU length of stay <24 h. (3) Missing values for platelet count, serum creatinine, or lactate dehydrogenase measured within the first 24 h of ICU admission. (4) Absence of outcome data.

### Data extraction

Patient data were collected during the first 24 h of ICU admission, including baseline demographics (age, sex, body mass index), severity-of-illness scores (Charlson Comorbidity Index, APACHE III, and SOFA), vital signs, comorbidities (myocardial infarction, congestive heart failure, stroke, malignancies, hepatic disease, metastatic tumors, and sepsis), therapeutic interventions (antimicrobial agents, vasopressors, mechanical ventilation, renal replacement therapy), and laboratory parameters (complete blood count, arterial blood gases, liver/renal function tests, coagulation studies). Primary outcomes included 28- and 90-day all-cause mortality and ICU length of stay. Covariates were selected if they met any of the following criteria: (1) *p* < 0.10 in univariate screening, (2) changed the effect estimate of EASIX on the outcome by >10% upon their addition to or removal from the model, or (3) were established confounders in prior literature. These variables, along with patient demographics, were included in the final multivariable model.

The Endothelial Activation and Stress Index (EASIX) was calculated as: (lactate dehydrogenase [U/L] × serum creatinine [mg/dL])/platelet count [10^9^/L].

### Missing data

No data were missing for primary outcome measures including lactate dehydrogenase (LDH), serum creatinine, platelet count, or mortality counts at 28, 60, and 90 days. The extent of missing data in the MIMIC-IV database is summarized below: body mass index (BMI) (42.14%), Sequential Organ Failure Assessment (SOFA) score (39.23%), basophils (34.20%), eosinophils (34.18%), monocytes (34.20%), lymphocytes (34.20%), neutrophils (34.18%), prothrombin time (PT) (7.4%), international normalized ratio (INR) (7.4%), activated partial thromboplastin time (aPTT) (7.54%), total bilirubin (TBIL) (38.39%), alanine aminotransferase (ALT) (38.67%), aspartate aminotransferase (AST) (38.07%), alkaline phosphatase (ALP) (38.67%), base excess (BE) (28.75%), calcium (6.23%), partial pressure of oxygen (PO_2_) (28.73%), partial pressure of carbon dioxide (PCO_2_) (28.75%), total CO_2_ (28.75%), PaO_2_/FiO_2_ ratio (47.85%), albumin (ALB) (53.99%), D-dimer (98.66%), fibrinogen (FIB) (72.13%), creatine kinase (CK) (63.42%), and creatine kinase-MB isoenzyme (CK-MB) (63.42%). All other variables had <5% missing data or were complete. In the Zigong database, missingness was as follows: temperature 77.5%, WBC 20.2%, RDW-SD 20.2%, PO_2_ 76.3%, lactate 19.3%, hemoglobin 20.2%, glucose 12.8%, CRP 72.4%, anion gap 19.3%; all other variables were <5% or complete. Following initial descriptive analysis, multiple imputation was performed for missing values, while variables with >45% missingness were excluded from analysis.

### Statistical analysis

Normality of continuous variables was assessed with the Kolmogorov–Smirnov test. Variables that followed a normal distribution are summarized as mean ± SD and compared by independent-samples *t* test; non-normally distributed variables are presented as median [interquartile range, IQR] and compared by the Mann–Whitney *U* test. EASIX was log_2_-transformed (log_2_EASIX) to achieve normality. Categorical data are reported as counts (percentages) and compared with the *χ*^2^ test.

Multivariable Cox proportional-hazards regression was used to evaluate the association between EASIX and in-hospital mortality. We used a sequential, multivariable modeling strategy in both datasets to verify the robustness of our findings. Subgroup analyses and forest plots were employed to examine potential interactions between EASIX and targeted population. We used restricted cubic splines (RCS) to characterize the non-linear relationship between EASIX and all-cause mortality, with likelihood-ratio tests to evaluate spline terms and overall curve shape. The proportional-hazards assumption was verified with Schoenfeld residuals; residual plots provided visual confirmation. Discrimination of EASIX for predicting in-hospital mortality in patients with COPD was quantified by receiver-operating characteristic (ROC) curve analysis, yielding the area under the curve (AUC), sensitivity, and specificity. The optimal cut-off was determined by maximizing the Youden index.

Software: R version 4.3.2 (R Foundation for Statistical Computing, Vienna, Austria)^1^ and Free Statistics software v2.1.

## Results

### Participants characteristics

**Figure 1:**
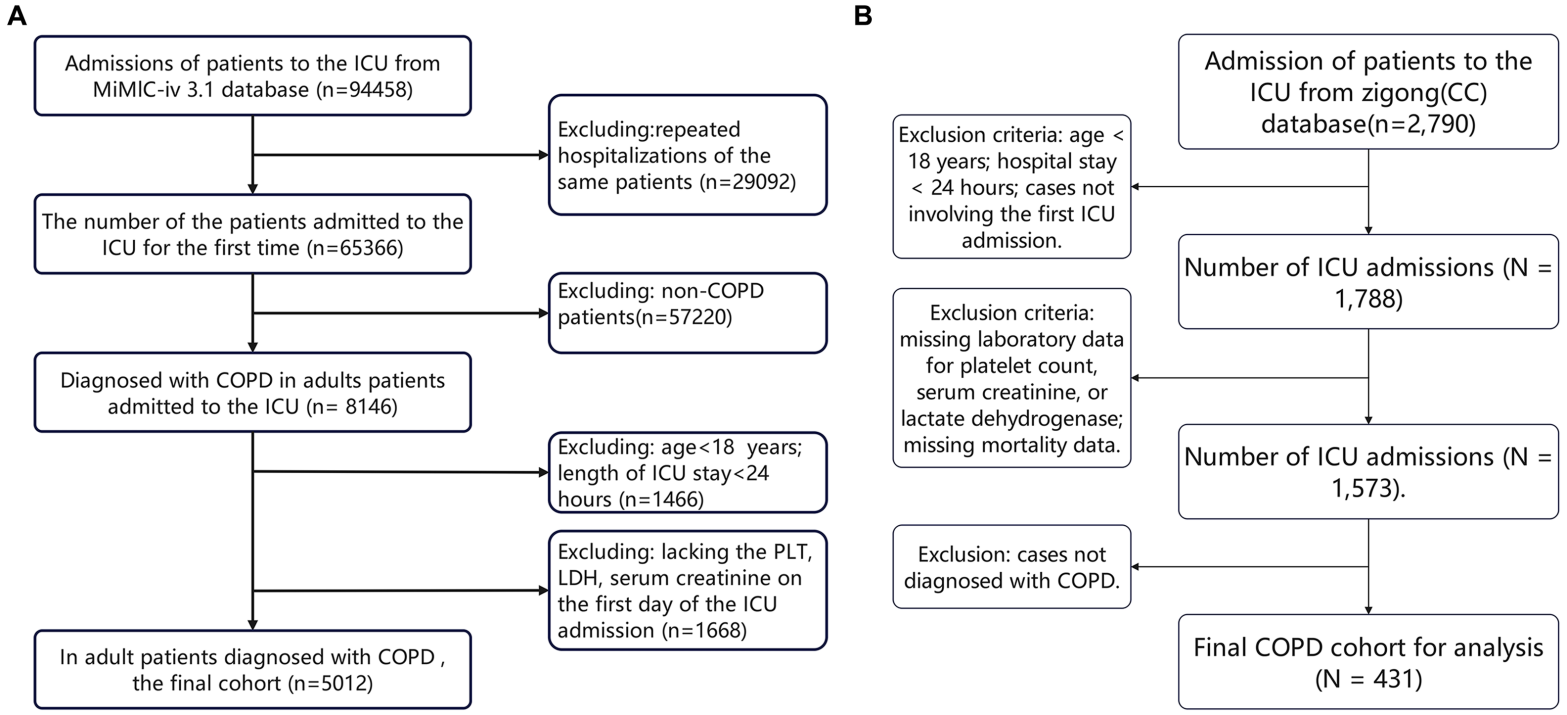
**(A, B)** Flow diagram of subject selection.

This study enrolled 5,012 and 431 eligible critically ill COPD patients from the MIMIC-IV 3.1 database and the Zigong database, respectively (Figures 1A,B). The former cohort comprised 2,755 (55%) men and 2,257 (45%) women, with a mean age of 71.4 ± 11.2 years; the latter included 328 (76.1%) men and 103 (23.9%) women, with a mean age of 75.9 ± 12.7 years. Across both cohorts, patients with higher EASIX values had markedly greater prevalences of heart failure, sepsis, and severe liver disease; were more likely to require vasopressors and mechanical ventilation; and exhibited elevated BUN, PT, APTT, AST, total bilirubin, lactate, and anion-gap levels. In MIMIC-IV, all-cause mortality rose steadily across increasing EASIX tertiles. In the Zigong cohort, however, mortality was similar between the lowest and middle tertiles (N1 and N2) but increased sharply in the highest tertile (N3), preserving an overall upward trend with higher EASIX values (Table 1).

**Table 1:** Baseline characteristics of the study populations.

Variables	MIMIC-IV database						Zigong database				
	Total (*n* = 5,012)	1 (*n* = 1,253)	2 (*n* = 1,253)	3 (*n* = 1,253)	4 (*n* = 1,253)	*p*	Total (*n* = 431)	1 (*n* = 144)	2 (*n* = 143)	3 (*n* = 144)	*p*
Gender, *n* (%)						<0.001					0.28
Female	2,257 (45.0)	710 (56.7)	567 (45.3)	491 (39.2)	489 (39)		103 (23.9)	41 (28.5)	30 (21)	32 (22.2)	
Male	2,755 (55.0)	543 (43.3)	686 (54.7)	762 (60.8)	764 (61)		328 (76.1)	103 (71.5)	113 (79)	112 (77.8)	
Age, Mean ± SD	71.4 ± 11.2	69.3 ± 11.4	71.9 ± 11.0	72.8 ± 10.7	71.4 ± 11.2	<0.001	75.9 ± 12.7	73.9 ± 12.3	75.2 ± 11.7	78.7 ± 13.6	0.004
Heart rate, Mean ± SD	85.8 ± 15.7	87.2 ± 15.4	85.3 ± 14.6	84.4 ± 16.1	86.5 ± 16.7	<0.001	93.4 ± 22.8	95.5 ± 23.0	92.5 ± 24.1	92.4 ± 21.1	0.441
MBP, Mean ± SD	77.3 ± 10.4	79.0 ± 10.8	77.8 ± 10.3	76.7 ± 10.2	75.6 ± 9.8	<0.001	84.6 ± 14.6	83.7 ± 16.1	83.2 ± 15.1	86.6 ± 12.7	0.471
SpO_2_, % Mean ± SD	96.2 ± 2.3	96.2 ± 2.1	96.2 ± 2.2	96.3 ± 2.2	96.1 ± 2.7	0.243	97.9 ± 5.7	98.0 ± 2.9	98.2 ± 2.9	97.6 ± 8.9	0.612
Ventilation, *n* (%)						<0.001					0.502
0	3,061 (61.1)	841 (67.1)	771 (61.5)	745 (59.5)	704 (56.2)		160 (37.1)	59 (41)	50 (35)	51 (35.4)	
1	1,951 (38.9)	412 (32.9)	482 (38.5)	508 (40.5)	549 (43.8)		271 (62.9)	85 (59)	93 (65)	93 (64.6)	
Vasopressin, *n* (%)						<0.001					<0.001
0	3,066 (61.2)	903 (72.1)	793 (63.3)	734 (58.6)	636 (50.8)		216 (50.1)	87 (60.4)	78 (54.5)	51 (35.4)	
1	1,946 (38.8)	350 (27.9)	460 (36.7)	519 (41.4)	617 (49.2)		215 (49.9)	57 (39.6)	65 (45.5)	93 (64.6)	
Diabetes, *n* (%)						<0.001					0.981
0	4,360 (87.0)	1,174 (93.7)	1,114 (88.9)	1,072 (85.6)	1,000 (79.8)		384 (89.1)	128 (88.9)	128 (89.5)	128 (88.9)	
1	652 (13.0)	79 (6.3)	139 (11.1)	181 (14.4)	253 (20.2)		47 (10.9)	16 (11.1)	15 (10.5)	16 (11.1)	
CHF, *n* (%)						<0.001					<0.001
0	2,730 (54.5)	869 (69.4)	697 (55.6)	598 (47.7)	566 (45.2)		294 (68.2)	118 (81.9)	99 (69.2)	77 (53.5)	
1	2,282 (45.5)	384 (30.6)	556 (44.4)	655 (52.3)	687 (54.8)		137 (31.8)	26 (18.1)	44 (30.8)	67 (46.5)	
Severe liver disease, *n* (%)						<0.001					0.011
0	4,794 (95.7)	1,240 (99)	1,231 (98.2)	1,204 (96.1)	1,119 (89.3)		324 (75.2)	119 (82.6)	108 (75.5)	97 (67.4)	
1	218 (4.3)	13 (1)	22 (1.8)	49 (3.9)	134 (10.7)		107 (24.8)	25 (17.4)	35 (24.5)	47 (32.6)	
Malignant cancer, *n* (%)						<0.001					0.653
0	4,203 (83.9)	1,000 (79.8)	1,071 (85.5)	1,097 (87.5)	1,035 (82.6)		399 (92.6)	131 (91)	134 (93.7)	134 (93.1)	
1	809 (16.1)	253 (20.2)	182 (14.5)	156 (12.5)	218 (17.4)		32 (7.4)	13 (9)	9 (6.3)	10 (6.9)	
Sepsis 3, *n* (%)						<0.001					<0.001
0	1,966 (39.2)	646 (51.6)	553 (44.1)	467 (37.3)	300 (23.9)		337 (78.2)	125 (86.8)	115 (80.4)	97 (67.4)	
1	3,046 (60.8)	607 (48.4)	700 (55.9)	786 (62.7)	953 (76.1)		94 (21.8)	19 (13.2)	28 (19.6)	47 (32.6)	
WBC, k/μl Median (IQR)	11.4 (8.4, 15.4)	11.6 (8.8, 15.7)	11.6 (8.7, 15.4)	11.2 (8.1, 15.2)	11.1 (8.0, 15.6)	0.005	11.8 ± 6.2	11.7 ± 6.0	12.0 ± 6.5	11.4 ± 6.2	0.791
HGB, g/dl Mean ± SD	10.6 ± 2.1	10.8 ± 1.9	10.9 ± 2.1	10.6 ± 2.1	10.2 ± 2.1	<0.001	119.1 ± 26.6	118.5 ± 25.2	122.9 ± 27.0	114.5 ± 27.6	0.074
PLT, k/μl Mean ± SD	211.9 ± 104.5	294.3 ± 115.6	217.3 ± 79.4	184.7 ± 78.4	151.4 ± 81.7	<0.001	163.9 ± 93.2	223.6 ± 92.9	172.1 ± 73.4	96.0 ± 62.0	<0.001
PT, Mean ± SD	16.3 ± 8.6	14.7 ± 5.9	15.3 ± 6.8	16.6 ± 9.2	18.4 ± 10.7	<0.001	14.9 ± 3.1	13.9 ± 1.7	14.2 ± 2.2	16.6 ± 4.2	<0.001
APTT, Mean ± SD	39.2 ± 19.6	35.4 ± 16.2	36.9 ± 17.2	40.0 ± 20.4	44.2 ± 22.6	<0.001	33.2 ± 13.1	31.3 ± 6.0	31.3 ± 10.9	37.0 ± 18.5	<0.001
INR, Mean ± SD	1.5 ± 0.8	1.3 ± 0.6	1.4 ± 0.7	1.5 ± 0.9	1.7 ± 1.0	<0.001	1.3 ± 0.3	1.2 ± 0.1	1.2 ± 0.2	1.4 ± 0.4	<0.001
ALB, g/dl Mean ± SD	3.2 ± 0.6	3.2 ± 0.6	3.3 ± 0.6	3.2 ± 0.6	3.1 ± 0.6	<0.001	3.3 ± 0.6	3.4 ± 0.6	3.5 ± 0.6	3.1 ± 0.6	<0.001
AST, μ/l Median (IQR)	36.5 (22.0, 78.1)	26.0 (18.0, 44.0)	32.0 (20.0, 55.0)	36.5 (22.0, 77.0)	59.0 (30.2, 181.0)	<0.001	32.5 (21.0, 56.2)	23.6 (18.1, 37.0)	32.5 (21.2, 49.1)	49.7 (28.8, 154.1)	<0.001
TBIL, mmol/l Median (IQR)	0.6 (0.4, 1.1)	0.4 (0.3, 0.7)	0.6 (0.3, 0.9)	0.6 (0.4, 1.1)	0.8 (0.4, 1.7)	<0.001	13.0 (9.0, 20.6)	12.1 (8.0, 16.9)	12.4 (8.9, 19.1)	16.9 (10.2, 27.7)	<0.001
BUN, mmol/l Median (IQR)	22.5 (15.5, 37.0)	15.5 (11.0, 21.0)	20.0 (14.5, 27.5)	25.0 (18.0, 39.0)	40.0 (27.0, 59.6)	<0.001	7.4 (5.6, 11.5)	6.2 (4.5, 8.3)	7.3 (5.8, 9.6)	11.0 (7.3, 16.9)	<0.001
SCR, mg/dl Median (IQR)	1.0 (0.8, 1.6)	0.7 (0.6, 0.9)	0.9 (0.8, 1.2)	1.2 (0.9, 1.6)	2.0 (1.4, 3.4)	<0.001	0.9 (0.7, 1.2)	0.6 (0.5, 0.8)	0.9 (0.7, 1.2)	1.3 (0.9, 2.2)	<0.001
AG, mmol/l Mean ± SD	14.3 ± 3.8	13.1 ± 3.0	13.5 ± 3.1	14.0 ± 3.5	16.5 ± 4.4	<0.001	12.5 ± 5.7	10.3 ± 3.9	12.1 ± 4.5	15.1 ± 7.1	<0.001
LAC, mmol/l Median (IQR)	1.7 (1.2, 2.5)	1.5 (1.1, 2.0)	1.6 (1.2, 2.2)	1.8 (1.3, 2.5)	2.0 (1.4, 3.3)	<0.001	1.9 (1.3, 2.7)	1.7 (1.2, 2.2)	1.8 (1.3, 2.7)	2.2 (1.5, 3.7)	<0.001
LDH, μ/l Median (IQR)	242.0 (190.0, 331.0)	192.0 (161.0, 229.0)	226.0 (186.0, 281.0)	265.0 (210.0, 344.0)	358.0 (256.0, 582.0)	<0.001	238.0 (189.0, 318.0)	199.5 (166.0, 242.5)	228.0 (194.0, 283.0)	323.5 (242.2, 575.2)	<0.001
mor_28d, *n* (%)						<0.001					<0.001
0	3,881 (77.4)	1,072 (85.6)	1,064 (84.9)	972 (77.6)	773 (61.7)		255 (59.2)	106 (73.6)	108 (75.5)	41 (28.5)	
1	1,131 (22.6)	181 (14.4)	189 (15.1)	281 (22.4)	480 (38.3)		176 (40.8)	38 (26.4)	35 (24.5)	103 (71.5)	
mor_90d, *n* (%)						<0.001					<0.001
0	3,476 (69.4)	975 (77.8)	972 (77.6)	863 (68.9)	666 (53.2)		223 (51.7)	97 (67.4)	98 (68.5)	28 (19.4)	
1	1,536 (30.6)	278 (22.2)	281 (22.4)	390 (31.1)	587 (46.8)		208 (48.3)	47 (32.6)	45 (31.5)	116 (80.6)	

Data presentation: normally distributed variables are represented as mean ± standard deviation (SD), skewed variables as median (IQR), and categorical variables as numbers (proportions). EASIX, endothelial activation and stress index; MBP, mean blood pressure; SpO_2_, saturation of peripheral oxygen; MI, myocardial infarct; CHF, congestive heart failure; CVD, cerebrovascular disease; CPD, chronic pulmonary disease; DM + CC, diabetes mellitus with chronic complications; MT, malignant tumor; MST, metastatic solid tumor; CRRT, continuous renal replacement therapy; IMV, invasive mechanical ventilation; apsiii, acute physiology score III; sofa, sequential organ failure assessment; TBIL, total bilirubin; LDH, lactate dehydrogenase; BG, blood glucose; BE, base excess; PaO_2_/FiO_2_, oxygenation index.

### Relationship between log_2_(EASIX) and mortality

Using the MIMIC-IV database, we built five sequential multivariable Cox models with normally distributed log_2_(EASIX) entered first as a raw continuous variable and then as tertile-based categorical covariates.

For 28-day mortality, continuous log2(EASIX) yielded hazard ratios (HRs) that were robust across all models: unadjusted HR 1.25 (95% CI 1.21–1.29, *p* < 0.001); adjusted HRs in Models 1–5: HRs ranged from 1.23 to 1.25 (all *p* < 0.001). In the fully adjusted Model 5, each one-unit increase in log2(EASIX) corresponded to a 23% higher mortality risk. When analyzed by tertiles, the highest tertile (vs. lowest) showed progressively attenuated yet significant HRs across models, decreasing from an unadjusted HR of 2.03 [95% CI 1.74–2.36, *p* < 0.001] to Model 5 HR of 1.66 [95% CI 1.39–1.98, *p* < 0.001].

A comparable pattern was observed for 90-day mortality (Table 2).

**Table 2:** Risk of 28-day and 90-day mortality according to Log2.EASIX in MIMIC.

Variable	Non-adjusted		Model 1		Model 2	
	Crude. HR (95%CI)	Crude. *p* value	Adj. HR (95%CI)	Adj. *p* value	Adj. HR (95%CI)	Adj. *p* value
28-day mortality						
Log2.EASIX	1.25 (1.21–1.29)	<0.001	1.25 (1.21–1.29)	<0.001	1.23 (1.19–1.27)	<0.001
Tertile of Log2.EASIX						
T1	Reference		Reference		Reference	
T2	1.31 (1.11–1.55)	0.002	1.24 (1.05–1.47)	0.011	1.25 (1.05–1.48)	0.012
T3	2.03 (1.74–2.36)	<0.001	1.98 (1.7–2.31)	<0.001	1.89 (1.62–2.22)	<0.001
90-day mortality						
Log2.EASIX	1.16 (1.13–1.2)	<0.001	1.16 (1.13–1.2)	<0.001	1.16 (1.12–1.19)	<0.001
Tertile of Log2.EASIX						
T1	Reference		Reference		Reference	
T2	1.26 (1.09–1.44)	0.001	1.18 (1.03–1.36)	0.019	1.21 (1.05–1.39)	0.008
T3	1.63 (1.43–1.84)	<0.001	1.57 (1.38–1.78)	<0.001	1.57 (1.37–1.79)	<0.001

Variable	Model 3		Model 4		Model 5	
	Adj. HR (95%CI)	Adj. *p* value	Adj. HR (95%CI)	Adj. *p* value	Adj. HR (95%CI)	Adj. *p* value
28-day mortality						
Log2.EASIX	1.23 (1.19–1.28)	<0.001	1.24 (1.19–1.29)	<0.001	1.23 (1.17–1.28)	<0.001
Tertile of Log2.EASIX						
T1	Reference		Reference		Reference	
T2	1.19 (1–1.42)	0.044	1.2 (1.01–1.43)	0.041	1.18 (0.99–1.4)	0.067
T3	1.74 (1.47–2.06)	<0.001	1.77 (1.49–2.1)	<0.001	1.66 (1.39–1.98)	<0.001
90-day mortality						
Log2.EASIX	1.18 (1.14–1.22)	<0.001	1.19 (1.15–1.23)	<0.001	1.18 (1.14–1.22)	<0.001
Tertile of Log2.EASIX						
T1	Reference		Reference		Reference	
T2	1.19 (1.03–1.37)	0.016	1.21 (1.05–1.39)	0.009	1.19 (1.03–1.38)	0.017
T3	1.58 (1.37–1.82)	<0.001	1.64 (1.42–1.89)	<0.001	1.55 (1.33–1.8)	<0.001

Model 1 adjusted for age, gender, BMI, heart rate, temperature, respiratory rate and SpO_2_. Model 2 adjusted for model 1 plus mycardial infarct, congestive heart failure, cerebrovascular disease, severe liver disease, chronic pulmonary disease, malignant cancer, metastatic solid tumor and sepsis. Model 3 adjusted for model 2 plus Mechanical ventilation, renal replacement therapy, use vasopressin, apsiii score and SOFA score. Model 4 adjusted for model 3 plus WBC, HGB, monocytes, neutrophils, APTT, INR and TBIL. Model 5 adjusted for model 3 plus lactate, aniongap, K+, Ca++, bicarbonate, PO_2_, PCO_2_, and total CO_2_. EASIX, endothelial activation and stress index; BMI, body mass index; SpO_2_, peripheral oxygen saturation; RRT, renal replacement therapy; APSIII, acute physiology score III; SOFA, sequential organ failure assessment; WBC, white blood cell; HGB, hemoglobin; APTT, activated partial thromboplastin time; INR, international normalized ratio; TBIL, total bilirubin; PO_2_, partial pressure of oxygen; PCO_2_, partial pressure of carbon dioxide.

In the Zigong cohort we constructed four sequential models to examine the same association. For 28-day mortality, continuous log_2_(EASIX) again demonstrated a significant positive association, with HRs progressing as follows: unadjusted HR 1.37 (95% CI 1.27–1.47, *p* < 0.001), adjusted HRs in Models 1–4 ranged from 1.32 to 1.43 (all *p* < 0.001).

When analyzed as tertiles, the highest-versus-lowest group comparison remained significant across all models, with HRs ranging from 3.05 (95% CI 2.10–4.42, *p* < 0.001) in the unadjusted analysis to 2.38 (95% CI 1.41–4.00, *p* = 0.001) in Model 4.

An identical pattern was observed for 90-day mortality (Table 3).

**Table 3:** Risk of 28-day and 90-day mortality according to Log2.EASIX in Zigong.

Variable	Non-adjusted		Model 1		Model 2	
	Crude. HR (95%CI)	Crude. *p* value	Adj. HR (95%CI)	Adj. *p* value	Adj. HR (95%CI)	Adj. *p* value
28-day mortality						
Log2.EASIX	1.37 (1.27–1.47)	<0.001	1.42 (1.31–1.53)	<0.001	1.43 (1.32–1.55)	<0.001
Tertile of Log2.EASIX						
T1	Reference		Reference		Reference	
T2	0.87 (0.55–1.38)	0.565	0.96 (0.6–1.55)	0.874	0.94 (0.58–1.51)	0.792
T3	3.05 (2.1–4.42)	<0.001	3.61 (2.41–5.41)	<0.001	3.66 (2.43–5.51)	<0.001
90-day mortality						
Log2.EASIX	1.35 (1.26–1.45)	<0.001	1.4 (1.3–1.5)	<0.001	1.42 (1.31–1.53)	<0.001
Tertile of Log2.EASIX						
T1	Reference		Reference		Reference	
T2	0.95 (0.63–1.43)	0.803	1.02 (0.67–1.57)	0.914	1.02 (0.66–1.56)	0.944
T3	2.97 (2.11–4.18)	<0.001	3.4 (2.35–4.91)	<0.001	3.42 (2.36–4.97)	<0.001

Variable	Model 3		Model 4	
	Adj. HR (95%CI)	Adj. *p* value	Adj. HR (95%CI)	Adj. *p* value
28-day mortality				
Log2.EASIX	1.33 (1.2–1.48)	<0.001	1.32 (1.17–1.5)	<0.001
Tertile of Log2.EASIX				
T1	Reference		Reference	
T2	0.82 (0.5–1.35)	0.442	0.94 (0.56–1.6)	0.824
T3	2.47 (1.55–3.94)	<0.001	2.38 (1.41–4)	0.001
90-day mortality				
Log2.EASIX	1.36 (1.24–1.5)	<0.001	1.32 (1.17–1.48)	<0.001
Tertile of Log2.EASIX				
T1	Reference		Reference	
T2	0.96 (0.62–1.49)	0.843	1.02 (0.63–1.65)	0.923
T3	2.75 (1.81–4.19)	<0.001	2.37 (1.47–3.81)	<0.001

Model 1 adjusted for age, gender, hypertension, diabetes, congestive heart failure, severe liver disease, renal failure, malignant cancer and sepsis. Model 2 adjusted for model 1 plus heart rate, MBP, respiratory rate, SpO_2_, Mechanical ventilation and use vasopressin. Model 3 adjusted for model 2 plus D-Dimer, Glu, WBC, HGB, PT, APTT, INR, RDWSD, LAC. Model 4 adjusted for model 3 plus SCR, AG, ALB, AST, BNP, GFR, TBIL, TNI, and UA. EASIX, endothelial activation and stress index; SpO_2_, peripheral oxygen saturation; MBP, mean blood Pressure; WBC, white blood cell; HGB, hemoglobin; GLU, glucose; PT, prothrombin time; APTT, activated partial thromboplastin time; INR, international normalized ratio; RDWSD, red cell distribution width - standard deviation; LAC, lactate; TBIL, total bilirubin; SCR, serum creatinine; AG, anion gap; ALB, albumin; AST, aspartate aminotransferase; BNP, B-type natriuretic peptide; GFR, glomerular filtration rate; TNI, troponin I; UA, uric acid.

These findings collectively confirm that log_2_(EASIX) is positively and independently associated with mortality in critically ill patients with COPD.

### Restricted cubic spline and inflection-point analysis

**Figure 2:**
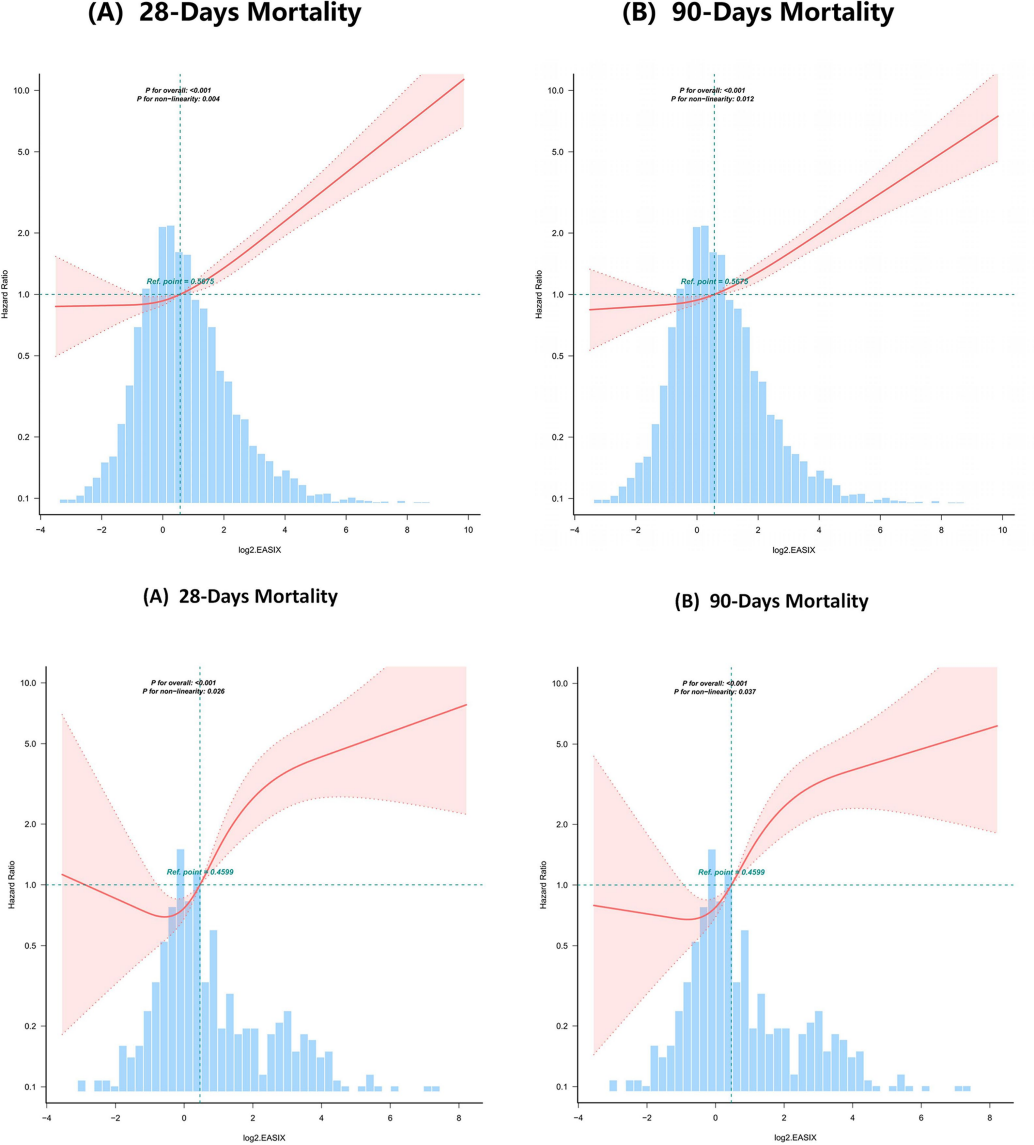
**(A, B)** Curve fitting of log2.EASIX and mortality. (Upper **AB**) Adjusted covariates included sex, age, BMI, vital signs, comorbidities, disease-severity scores, clinical interventions, and laboratory indices—complete blood count, liver function, coagulation profile, and arterial blood gas analysis. (Lower **AB**) Adjusted covariates included sex, age, vital signs, comorbidities, clinical interventions, and laboratory indices—complete blood count, liver function, renal function, coagulation profile, and arterial blood gas analysis.

Restricted cubic spline (RCS) analysis, adjusted for baseline characteristics, comorbidities, critical illness severity scores, ICU interventions, and key laboratory parameters, revealed a significant nonlinear dose–response relationship between log_2_(EASIX) and mortality in COPD patients. In the MIMIC-IV cohort, this nonlinearity was evident for both 28-day mortality (*p*-overall < 0.001, *p*-non-linearity = 0.004) and 90-day mortality (*p*-overall < 0.001, *p*-non-linearity = 0.012 (Figure 2A). Similar patterns were observed in the Zigong cohort for 28-day mortality (*p*-overall < 0.001, p-non-linearity = 0.026 and 90-day mortality (*p*-overall < 0.001, *p*-non-linearity = 0.037; Figure 2B).

Breakpoint analysis confirmed a threshold effect in MIMIC-IV (likelihood-ratio *p* < 0.05, Table 4). Using a two-segment Cox model with the knot set at log_2_(EASIX) ≈ 3.49, the hazard ratios below the knot were 1.168 (95% CI: 1.100–1.239, *p* < 0.001) for 28-day mortality and 1.144 (1.088–1.202, *p* < 0.001) for 90-day mortality; above the knot, these HRs increased to 1.805 (1.367–2.383, *p* < 0.001) and 1.597 (1.219–2.092, *p* < 0.001), respectively. In the Zigong cohort, the likelihood-ratio test for a threshold effect was non-significant (*p* > 0.05, Table 5). Nevertheless, exploratory piecewise analysis with a knot between log_2_(EASIX) 3.42 and 3.70 revealed HRs below the knot of 1.823 (95% CI: 1.532–2.171, *p* < 0.001) for 28-day and 1.78 (1.501–2.112, *p* < 0.001) for 90-day mortality; above the knot the HRs decreased to 0.04 (0.004–0.399, *p* = 0.006) and 0.272 (0.087–0.856, *p* = 0.026), respectively.

**Table 4:** Threshold effect analysis of the relationship between Log2.EASIX and all-cause mortality in MIMIC.

Log2.EASIX	HR (95%CI)	*p* value
28 Days mortality		
<3.492	1.168 (1.1, 1.239)	<0.001
≥3.492	1.805 (1.367, 2.383)	<0.001
Likelihood ratio test		0.001
90 Days mortality		
<3.493	1.144 (1.088, 1.202)	<0.001
≥3.493	1.597 (1.219, 2.092)	<0.001
Likelihood ratio test		0.012

The upper limit for the X-axis values is capped at 99.5%.

**Table 5:** Threshold effect analysis of the relationship between Log2.EASIX and all-cause mortality in Zigong.

Log2.EASIX	HR (95%CI)	*p* value
28 Days mortality		
<3.695	1.823 (1.532, 2.171)	<0.001
≥3.695	0.04 (0.004, 0.399)	0.0061
Likelihood ratio test		0.282
90 Days mortality		
<3.416	1.78 (1.501, 2.112)	<0.001
≥3.416	0.272 (0.087, 0.856)	0.0261
Likelihood ratio test		0.259

The upper limit for the X-axis values is capped at 99.5%.

### Proportional hazards assumption testing

The proportional-hazards assumption for EASIX was verified in both datasets. In MIMIC-IV, the Schoenfeld test for EASIX yielded *p* = 0.319, and the global test gave *χ*^2^ = 25.925 (df = 23, *p* = 0.304) (Supplementary Table 1). In the Zigong cohort, the corresponding values were *p* = 0.485 for EASIX and *χ*^2^ = 24.12 (df = 18, *p* = 0.151) (Supplementary Table 2). Schoenfeld residual plots further confirmed these findings (Supplementary Figures S1, S2).

### Subgroup analysis and forest plot

**Figure 3:**
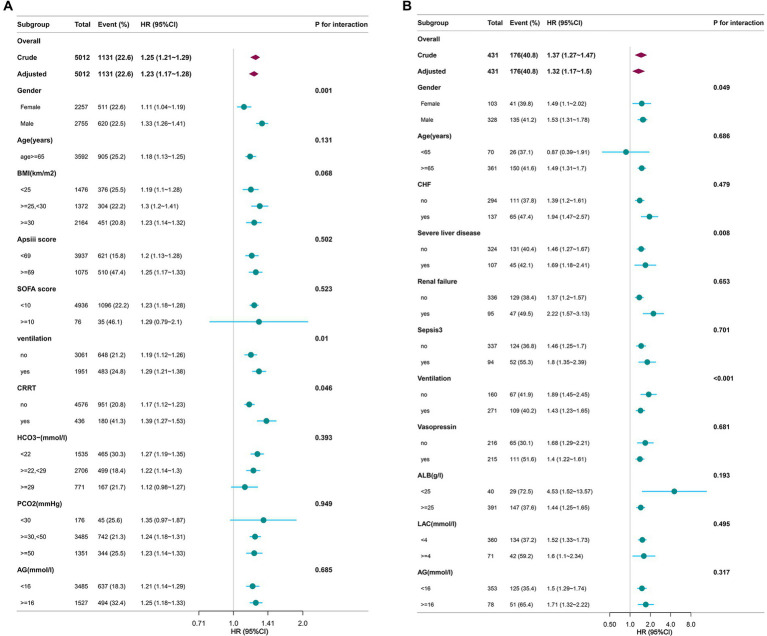
**(A, B)** Forest plot of Log2.EASIX and covariates. BMI, body mass index; CRRT, continuous renal replacement therapy; APSIII, acute physiology score III; SOFA, sequential organ failure assessment; AG, anion gap; EASIX, endothelial activation and stress index; CHF, congestive heart failure; ALB, albumin; LAC, lactate; AG, anion gap.

Figures 3A,B summarizes the subgroup analyses. After stratifying by baseline characteristics, severity-of-illness scores, ICU interventions, complete blood counts, arterial blood gas values, and other key laboratory parameters, in MIMIC-IV database we observed statistically significant interactions for sex (*p* = 0.001), mechanical ventilation (*p* = 0.010) and renal-replacement therapy (*p* = 0.046). In the Zigong dataset, significant effect modification was observed for sex (*p* = 0.049), mechanical ventilation (*p* < 0.001) and severe liver disease (*p* = 0.008).

### Receiver operating characteristic analysis

**Figure 4:**
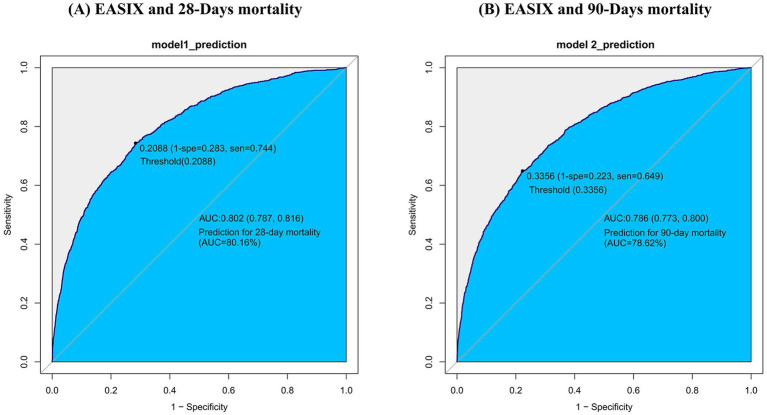
ROC curve of EASIX and mortality in MIMIC.

**Figure 5:**
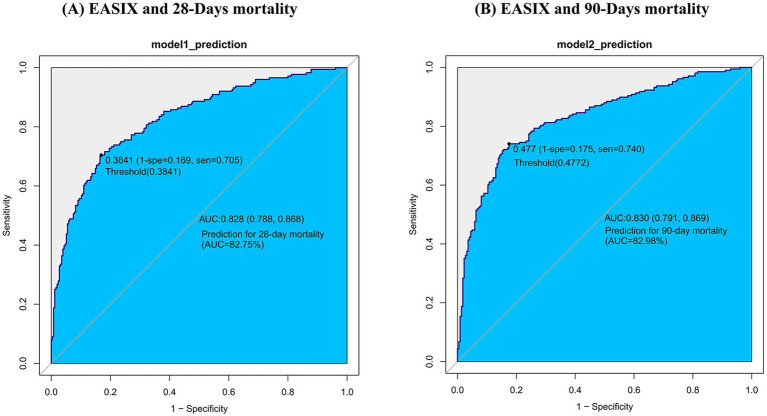
ROC curve of EASIX and mortality in Zigong.

We assessed EASIX’s ability to predict all-cause mortality at 28 and 90 days in this COPD cohort using area under the receiver operating characteristic curve (AUC) analysis. In the MIMIC-IV cohort, the AUROC of EASIX for 28-day and 90-day mortality was 80.2 and 78.6%, respectively (Figure 4). At the optimal cut-offs, the two metrics achieved Youden indices of 0.4602 and 0.4264 (Table 6). In the Zigong cohort, EASIX yielded an AUROC of 82.8% for 28-day mortality and 83.0% for 90-day mortality (Figure 5). At the optimal cut-points, the two metrics posted Youden indices of 0.536 and 0.565 (Table 7).

**Table 6:** Information of receiver operating characteristic curve in Figure 4.

Variable	AUC	95%CI	Threshold	Sensitivity	Specificity	Youden
prediction for 28 days	80.1598%	78.7008–81.6187%	0.2088	0.7436	0.7166	0.4602
prediction for 90 days	78.6427%	77.2814–80.004%	0.3356	0.6491	0.7773	0.4264

AUC, area under the curve.

**Table 7:** Information of receiver operating characteristic curve in Figure 5.

Variable	AUC	95%CI	Threshold	Sensitivity	Specificity	Youden
prediction for 28 days	82.754%	78.7534–86.7547%	0.3841	0.7045	0.8314	0.5359
prediction for 90 days	82.9812%	79.0715–86.8909%	0.4772	0.7404	0.8251	0.5655

AUC, area under the curve.

## Discussion

In this real-world cohort study, we demonstrated a positive, dose-dependent association between EASIX and all-cause mortality in critically ill patients with COPD. This association remained robust and consistent after extensive adjustment for confounders and across multiple time-to-event models. Subgroup analyses revealed no meaningful interactions that would compromise these findings. Sensitivity analyses using log_2_ (EASIX) tertiles confirmed a dose-dependent risk gradient, with the highest tertile exhibiting the most pronounced hazard ratios. Moreover, EASIX exhibits a clear threshold effect, with mortality risk changing significantly once the cut-point is crossed. Proportional hazards testing showed that the effect of EASIX on 28-day mortality was constant over time, obviating the need to treat it as a time-varying covariate. The global test further indicated that the proportional-hazards assumption was satisfied for the overall model, validating the use of the Cox proportional-hazards model. Finally, we evaluated the predictive performance of log_2_(EASIX); both the AUROC and Youden index indicated good discriminative ability.

Prior studies have focused on developing composite biomarker indices to predict outcomes in COPD. These indices primarily incorporate peripheral blood components, including the neutrophil-to-albumin ratio, neutrophil-to-lymphocyte ratio, and eosinophil-to-lymphocyte ratio (23). These studies establish disease associations through inflammatory mechanisms, lending them considerable persuasive power. Their limitation, however, is that all data derive from a single peripheral-blood inflammatory panel (24); the authors themselves acknowledge the absence of comparative markers such as interleukin-6 and procalcitonin. Consequently, the panel’s ability to accurately characterize the inflammatory state may be suboptimal (25). The present study examines COPD and further extends the research paradigm by incorporating the EASIX score, which integrates lactate dehydrogenase (LDH), serum creatinine, and platelet levels. LDH is a ubiquitous cytosolic enzyme present in cardiomyocytes, skeletal muscle, hepatocytes, endothelial cells, and erythrocytes; its elevation reflects cellular injury and tissue hypoxia. Serum creatinine serves as a biomarker of renal dysfunction attributable to microvascular compromise and impaired renal perfusion. Platelet count conveys information on coagulation status and endothelial homeostasis. By synthesizing these multi-system parameters, the EASIX score may offer broader clinical utility in reflecting the body’s overall stress level or endothelial injury. Previous studies have examined the association between EASIX and chronic pulmonary disorders in asthmatic patients (26); this study extends the findings by demonstrating its applicability to COPD.

In our dataset, the fully adjusted model (model 5) in Table 2 yielded a *p*-value of 0.67 for the T2 group in relation to 28-day mortality. Although this value is non-significant, it does not negate the predictive utility of EASIX: the hazard ratio remains directionally consistent with the overall trend, and the lower bound of the 95% CI is still close to 1.0. The absence of statistical significance may reflect the modest difference in the number of deaths between the T1 and T2 groups. Moreover, several covariates adjusted for in model 5—most notably lactate and arterial blood gas parameters—may act as mediators on the causal pathway linking EASIX to mortality. Endothelial activation elevates LDH, which in turn increases circulating lactate (27); elevated lactate widens the anion gap (28), and a widened anion gap is associated with higher COPD-related mortality (29). These mediators therefore may largely explain the observed association. After evaluating the potential for overfitting, we nevertheless present the sensitivity analyses to ensure full transparency.

For the inflection-point analysis, we employed the same covariate set used in the multivariable models (Table 2) to adjust for potential confounders. Log_2_(EASIX) truncated at the 99.5th-percentile, was selected to enhance normality and mitigate the impact of extreme values. In the MIMIC-IV cohort, log_2_(EASIX) showed highly consistent predictive performance for both 28- and 90-day mortality, with inflection points consistently observed at log_2_(EASIX) ≈ 3.5–3.7 (raw EASIX ≈ 11–13). Below this threshold, hazards remained stable (HR ≈ 1.14–1.17), while a sharp increase occurred above it (HR ≈ 1.6–1.8). Clinically, the log_2_(EASIX) ≈ 3.5 (raw EASIX ≈ 11.3) inflection represents a high-risk threshold: patients with EASIX > 11 had an 80.5% higher 28-day mortality risk (HR = 1.805) versus those with EASIX ≤ 11.

In the Zigong cohort the sequence of findings was as follows: the preceding RCS analysis had already established that the relation is not linear. Second, the likelihood-ratio test for the piece-wise (two-segment) linear model yielded *p* > 0.05, indicating merely that this specific “bent-line” form of non-linearity offers no statistically significant advantage over the smooth RCS curve. This in no way negates the existence of a non-linear relationship. Non-linear Test*2 (*p* = 0.049 and 0.072)—one significant and one borderline—further indicates that the slopes of the two segments differ, i.e., the curve truly “bends.” The pattern resembles an inverted U: a steep upswing, a peak at the inflection point, followed by a sharp downslope. This shape was highly consistent across both 28-day and 90-day outcomes.

Yet this inverted-U pattern may not represent a genuine biological phenomenon; it could stem from: (1) Survivor bias—the most likely explanation. Only a highly selected subset of patients lives long enough to reach extreme EASIX values; those with fulminant illness often die before such levels can be measured. Individuals who survive to the point of registering ultra-high values are, by definition, “survival champions,” and their subsequent mortality is therefore lower; 2. Data sparsity—the region to the right of the knot contains very few ultra-high EASIX observations, producing unstable estimates. Although the hazard ratios are statistically significant, their confidence intervals are extremely wide (e.g., 0.004–0.399 and 0.087–0.856), reflecting this uncertainty; 3. Potential confounding by treatment interventions cannot be ruled out; 4. It is possible that disease heterogeneity—stemming from the various phenotypes of severe COPD—may have affected our findings. The findings from the Zigong cohort should be considered exploratory and hypothesis-generating, warranting validation in future studies.

Taken together, MIMIC-IV and Zigong data concur that the relation between EASIX and mortality is non-linear. The contrasting patterns—an “inverted U-shaped” risk relationship in the Zigong cohort versus a more stable threshold effect observed in MIMIC-IV—likely reflect differences in sample size and statistical power between the two cohorts.

In the forest-plot interaction analysis, the association between EASIX and mortality was significantly stronger in men (*p* for interaction = 0.001, *p* for interaction = 0.049). Plausible explanatory mechanisms include: (1) estrogen-mediated anti-inflammatory and endothelial-protective effects in women—which are hypothesized to act partly through enhanced nitric oxide (NO) production (30) that may attenuate endothelial injury-related mortality (31), contrasting with androgen-potentiated endothelial activation in men; (2) men’s higher predisposition to smoking-related COPD and its frequent cardiovascular comorbidities (32), which synergize with endothelial dysfunction to amplify mortality risk; and 3. clinical observations that male patients typically present later and exhibit poorer treatment adherence, factors that may exacerbate the mortality impact at elevated EASIX levels. Second, the association between EASIX and mortality was significantly stronger in mechanically ventilated patients (*p* for interaction = 0.01, *p* for interaction <0.001), potentially due to three interrelated mechanisms: (1) ventilator-induced barotrauma directly damages pulmonary vascular endothelium (33), synergizing with EASIX-reflected systemic endothelial activation to accelerate multi-organ failure; (2) the characteristic hypoxemia and systemic inflammation (e.g., elevated interleukin-6) in COPD patients requiring ventilation further amplify endothelial activation (33), thereby enhancing EASIX’s prognostic value; (3) and pre-existing vascular leak in severe COPD exacerbations (33) suggests that extensive endothelial disruption (indicated by high EASIX) may become a particularly lethal combination in this vulnerable population. Third, the stronger association between EASIX and mortality in CRRT patients (*p* for interaction = 0.046) may be explained through three pathophysiological mechanisms: (1) Acute kidney injury (AKI)—a near-universal condition in CRRT populations—not only directly impairs endothelial function through uremic toxins (34), and synergizes with systemic inflammation and microthrombosis to increase mortality (35), but is also reflected in high EASIX scores; (2) Circuit-related factors including membrane biocompatibility and anticoagulation regimens may paradoxically exacerbate endothelial activation, compounding EASIX-associated risks; and (3) CRRT itself may preserve pathogenic endothelial activators (particularly complement fragments C3b and C5a) (36) that may amplify EASIX’s predictive capacity for adverse outcomes. An interaction was observed between EASIX and CRRT treatment in the MIMIC-IV cohort (likely reflecting an interaction with the AKI population), whereas no such interaction was found with chronic renal failure in the Zigong cohort. Given that serum creatinine is a core component of the EASIX score, we propose that EASIX captures the overlapping domains of endothelial stress and acute kidney injury. Finally, the association between EASIX and mortality was significantly stronger among patients with severe liver disease (*p* for interaction = 0.008). Prior studies have shown that endothelial dysfunction in acute or chronic liver failure is accompanied by elevated EASIX values (37), supporting the use of this index to prognosticate in advanced liver disease. Our findings demonstrate that, within the specific subgroup of critically ill COPD patients who also have severe hepatic impairment, higher EASIX levels confer an even greater risk of death—an observation that aligns perfectly with the previously proposed pathophysiologic mechanism.

Regarding the coupling of lactate dehydrogenase (LDH), serum creatinine, and platelets in the EASIX score, a possible mechanistic explanation is as follows: LDH serves as a marker of cellular injury, with elevated levels reflecting endothelial barrier disruption as vascular endothelial cells contain abundant LDH. This endothelial damage exposes the basement membrane, triggering platelet activation and aggregation. Subsequent release of inflammatory mediators promotes microthrombus formation, which may obstruct renal tubules and impair renal microcirculation—ultimately manifesting as elevated serum creatinine levels. Integrating the mechanistic interpretations from our experimental data, elevated EASIX likely reflects chronic airway inflammation-induced endothelial dysfunction through multiple pathways: (1) inflammatory mediators (including TNF-*α*, IL-1β, and IL-6) directly damage endothelial cells; (2) activation of Toll-like receptors (TLRs) and the NLRP3 inflammasome drives endothelial dysfunction; and (3) NF-κB signaling pathway activation upregulates endothelial adhesion molecules (particularly VCAM-1 and ICAM-1) (6). This pattern contrasts with EASIX’s association with thrombotic microangiopathy in hematologic disorders, highlighting disease-specific endothelial injury mechanisms (16). Current evidence demonstrates elevated IL-33/ST2 pathway activity in COPD, originating from both airway epithelial cells and peripheral blood monocytes. This pathway contributes to systemic inflammation by promoting inflammatory cell infiltration, exacerbating airway inflammation, and increasing vascular endothelial permeability—thereby worsening airway obstruction (38). Furthermore, chronic hypoxemia in COPD induces hypoxia-inducible factor-1α (HIF-1α) release, which directly mediates endothelial dysfunction (8). Together, these observations constitute a rationale for the association of COPD with endothelial injury.

Several limitations should be acknowledged. First, key confounders such as smoking status, GOLD stage, corticosteroid use, and socioeconomic status were unavailable in either database and could not be adjusted for. Second, certain variables—including BMI, CRP, albumin, PaO_2_/FiO_2_ ratio, and D-dimer—were available in only one of the two cohorts. Although multiple imputation was performed, this may have introduced residual confounding and selection bias, potentially leading to an overestimation of the true effect sizes. Third, some variables with high missingness rates (e.g., CRP, with 72.39% missing) were ultimately excluded from the analysis. The exclusion of these inflammatory and nutritional indicators may have further amplified the relative strength of the EASIX score in our models, as it may have partially absorbed their predictive effects. Our interaction mechanisms rely on literature rather than direct experimental evidence as we lacked measurements of sex hormones, endothelial function markers, or inflammatory cytokines. Future prospective studies should serially monitor EASIX with these molecular parameters to clarify their dynamic relationships with outcomes. Because the inflection points were identified solely through statistical algorithms, the two cohorts exhibit different curve shapes; the clinical relevance of these patterns requires validation in external populations.

## Conclusion

Among COPD patients admitted to the ICU, EASIX showed a nonlinear, positive association with both 28- and 90-day all-cause mortality, with death risk rising progressively as the index increased. EASIX demonstrates strong utility for early risk stratification in critically ill COPD patients and offers good performance for short- to intermediate-term prognostication.

## Data Availability

The raw data supporting the conclusions of this article will be made available by the authors, without undue reservation.

## Footnotes

1
https://www.r-project.org

## Supplementary material

SUPPLEMENTARY FIGURE S1 Residual plot (EASIX and 28-day mortality) in mimic.

SUPPLEMENTARY FIGURE S2 Residual plot (EASIX and 28-day mortality) in Zigong.
