# Supplementary figures and images for: Association between the Endothelial Activation and Stress Index and all-cause mortality in patients with chronic obstructive pulmonary disease
Source: Front Med (Lausanne). 2026 May 13;13:1732176. doi: 10.3389/fmed.2026.1732176 (PMC13212528; doi:10.3389/fmed.2026.1732176)

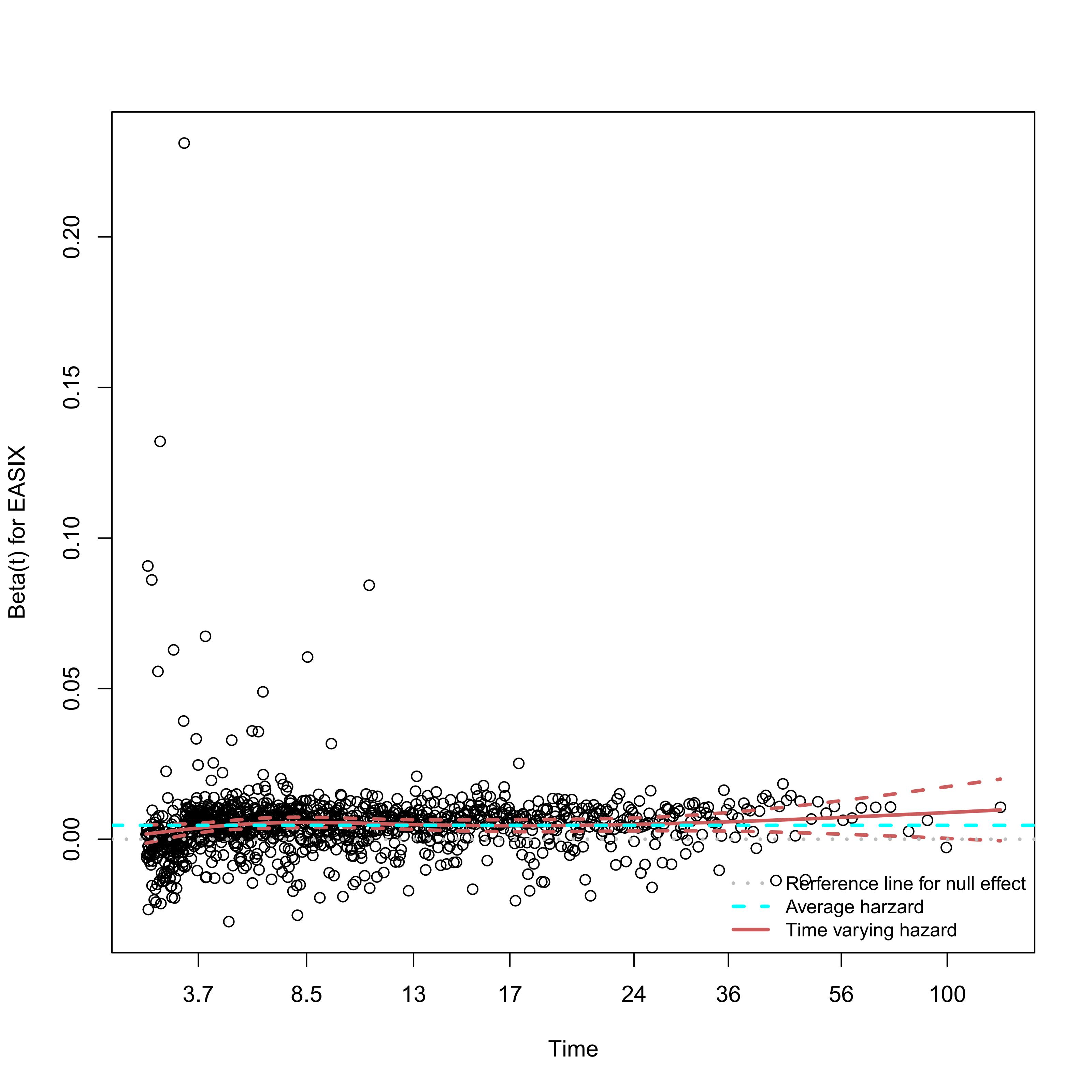

Supplement: SUPPLEMENTARY FIGURE S1 — Residual plot (EASIX and 28-day mortality) in mimic. [file Image_1.jpeg]

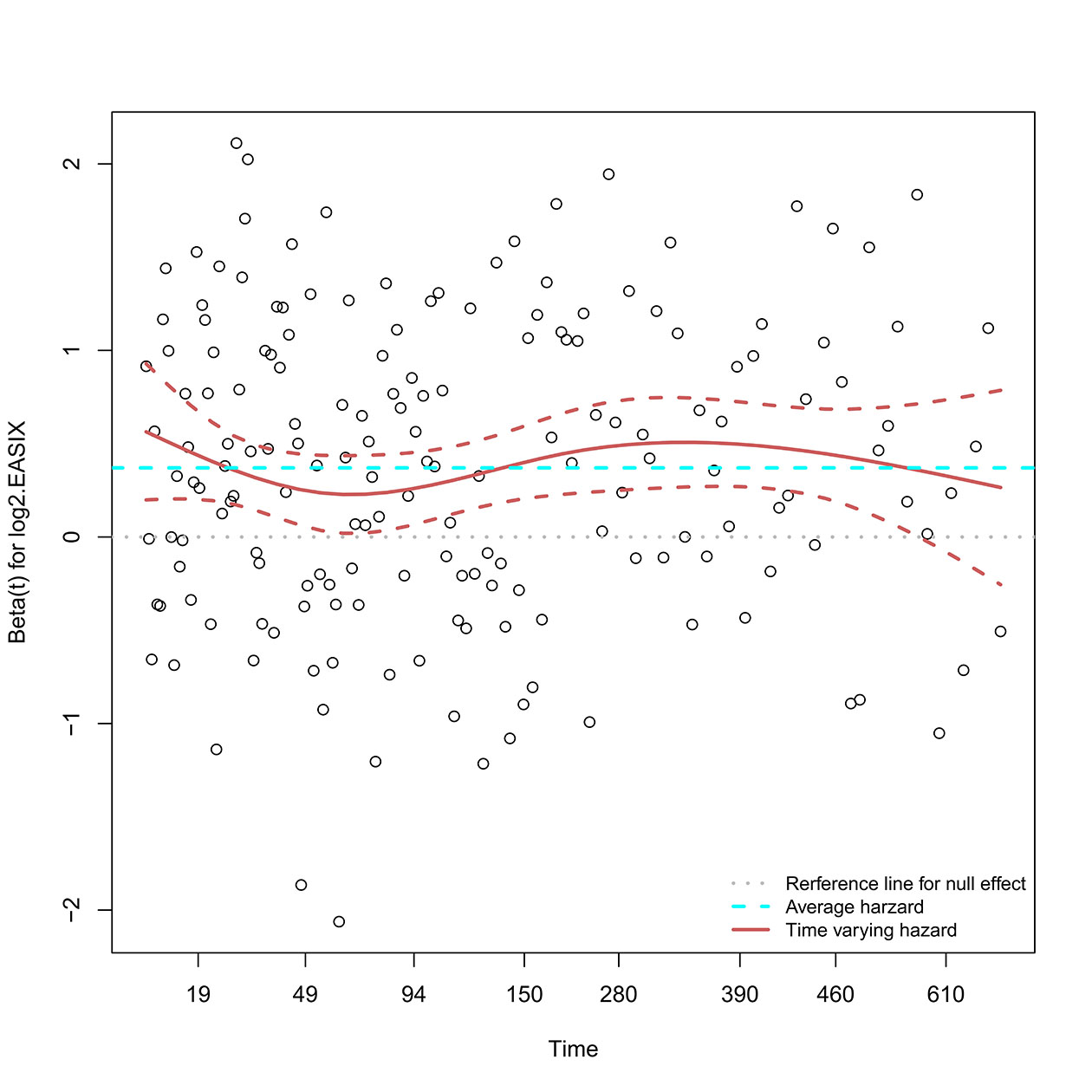

Supplement: SUPPLEMENTARY FIGURE S2 — Residual plot (EASIX and 28-day mortality) in Zigong. [file Image_2.jpeg]
