# Supplementary material for: Association between the Endothelial Activation and Stress Index and all-cause mortality in patients with chronic obstructive pulmonary disease
Source: Front Med (Lausanne). 2026 May 13;13:1732176. doi: 10.3389/fmed.2026.1732176 (PMC13212528; doi:10.3389/fmed.2026.1732176)
Supplement: Supplementary file 3 [file Table_1.docx]

**Supplementary table 1: The proportional hazards model variable test for the relationship between EASIX and 28-day mortality rate model**

| Variable | chisq | df | p.value |
| --- | --- | --- | --- |
| EASIX | 0.993 | 1 | 0.319 |
| gender | 1.837 | 1 | 0.175 |
| admission_age | 2.337 | 1 | 0.126 |
| BMI_value | 2.133 | 1 | 0.144 |
| spo2_mean | 3.001 | 1 | 0.083 |
| temperature_mean | 0.306 | 1 | 0.58 |
| myocardial_infarct | 0.066 | 1 | 0.797 |
| cerebrovascular_disease | 0.092 | 1 | 0.762 |
| malignant_cancer | 1.08 | 1 | 0.299 |
| metastatic_solid_tumor | 0.822 | 1 | 0.365 |
| severe_liver_disease | 2.36 | 1 | 0.124 |
| crrt_present | 1.236 | 1 | 0.266 |
| wbc_mean | 0.014 | 1 | 0.905 |
| hemoglobin_mean | 0.823 | 1 | 0.364 |
| abs_monocytes_mean | 0 | 1 | 0.995 |
| abs_neutrophils_mean | 0.275 | 1 | 0.6 |
| aptt_mean | 1.754 | 1 | 0.185 |
| inr_mean | 0.757 | 1 | 0.384 |
| TBIL_mean | 1.677 | 1 | 0.195 |
| potassium_mean | 3.364 | 1 | 0.067 |
| calcium_mean | 0.747 | 1 | 0.387 |
| po2_mean | 0.743 | 1 | 0.389 |
| pco2_mean | 0.408 | 1 | 0.523 |
| GLOBAL | 25.925 | 23 | 0.304 |

**Note**:EASIX and other variables all meet the proportional hazards (PH) assumption. This suggests that the main variables in the model have a stable impact on mortality risk within 28 days (the hazard ratio does not change over time). The global test indicates that the overall model meets the PH assumption, showing that the model is time-independent and robust for use.
