# Supplementary material for: Association between the Endothelial Activation and Stress Index and all-cause mortality in patients with chronic obstructive pulmonary disease
Source: Front Med (Lausanne). 2026 May 13;13:1732176. doi: 10.3389/fmed.2026.1732176 (PMC13212528; doi:10.3389/fmed.2026.1732176)
Supplement: Supplementary file 5 [file Table_3.docx]

**Supplementary Table 3.** Risk of 28-day and 90-day Mortality according to Log2.EASIX.(adjusted lac and anion gap)

| **Variable** | **Non-adjusted** | | **Model 1** | | **Model 2** | |
| --- | --- | --- | --- | --- | --- | --- |
|  | **crude.HR (95%CI)** | **crude.P value** | **adj.HR (95%CI)** | **adj.P value** | **adj.HR (95%CI)** | **adj.P value** |
| **28-day mortality** |  |  |  |  |  |  |
| Log2.EASIX | 1.37 (1.27~1.47) | <0.001 | 1.42 (1.31~1.53) | <0.001 | 1.43 (1.32~1.55) | <0.001 |
| Tertile of Log2.EASIX |  |  |  |  |  |  |
| T1 | reference |  | reference |  | reference |  |
| T2 | 0.87 (0.55~1.38) | 0.565 | 0.96 (0.6~1.55) | 0.874 | 0.94 (0.58~1.51) | 0.792 |
| T3 | 3.05 (2.1~4.42) | <0.001 | 3.61 (2.41~5.41) | <0.001 | 3.66 (2.43~5.51) | <0.001 |
| **90-day mortality** |  |  |  |  |  |  |
| Log2.EASIX | 1.35 (1.26~1.45) | <0.001 | 1.4 (1.3~1.5) | <0.001 | 1.42 (1.31~1.53) | <0.001 |
| Tertile of Log2.EASIX |  |  |  |  |  |  |
| T1 | reference |  | reference |  | reference |  |
| T2 | 0.95 (0.63~1.43) | 0.803 | 1.02 (0.67~1.57) | 0.914 | 1.02 (0.66~1.56) | 0.944 |
| T3 | 2.97 (2.11~4.18) | <0.001 | 3.4 (2.35~4.91) | <0.001 | 3.42 (2.36~4.97) | <0.001 |
| **Variable** | **Model 3** | | **Model 4** | |  |  |
|  | **adj.HR (95%CI)** | **adj.P value** | **adj.HR (95%CI)** | **adj.P value** |  |  |
| **28-day mortality** |  |  |  |  |  |  |
| Log2.EASIX | 1.33 (1.2~1.48) | <0.001 | 1.34 (1.19~1.52) | <0.001 |  |  |
| Tertile of Log2.EASIX |  |  |  |  |  |  |
| T1 | reference |  | reference |  |  |  |
| T2 | 0.85 (0.52~1.39) | 0.509 | 0.93 (0.55~1.57) | 0.783 |  |  |
| T3 | 2.63 (1.66~4.17) | <0.001 | 2.49 (1.48~4.17) | 0.001 |  |  |
| **90-day mortality** |  |  |  |  |  |  |
| Log2.EASIX | 1.37 (1.24~1.51) | <0.001 | 1.34 (1.19~1.5) | <0.001 |  |  |
| Tertile of Log2.EASIX |  |  |  |  |  |  |
| T1 | reference |  | reference |  |  |  |
| T2 | 0.99 (0.63~1.53) | 0.948 | 1.01 (0.62~1.62) | 0.982 |  |  |
| T3 | 2.93 (1.93~4.44) | <0.001 | 2.48 (1.54~3.98) | <0.001 |  |  |

**Model 1** adjusted for age, gender,hypertension, diabetes, congestive heart failure, severe liver disease, renal failure, malignant cancer and sepsis.

**Model 2** adjusted for model 1 plus heart rate, MBP, respiratory rate, Spo2, Mechanical ventilation and use vasopressin.

**Model 3** adjusted for model 2 plus D-Dimer, Glu, WBC, HGB, PT, APTT, INR, RDWSD.

**Model 4** adjusted for model 3 plus SCR, ALB, AST, BNP, GFR, TBIL, TNI and UA .

**Abbreviations**: EASIX,Endothelial Activation and Stress Index; Spo2,Peripheral Oxygen Saturation; MBP, Mean Blood Pressure; WBC,White Blood Cell ;HGB, hemoglobin; GLU, Glucose; PT, Prothrombin Time ; APTT,Activated Partial Thromboplastin Time; INR, International Normalized Ratio; RDWSD, Red Cell Distribution Width - Standard Deviation; LAC, Lactate; TBIL, Total Bilirubin; SCR,Serum Creatinine ;AG, Anion Gap; ALB, Albumin; AST, Aspartate Aminotransferase; BNP, B-type Natriuretic Peptide; GFR, Glomerular Filtration Rate; TNI, Troponin I; UA, Uric Acid.
